# Loqusdb: added value of an observations database of local genomic variation

**DOI:** 10.1186/s12859-020-03609-z

**Published:** 2020-07-01

**Authors:** Måns Magnusson, Jesper Eisfeldt, Daniel Nilsson, Adam Rosenbaum, Valtteri Wirta, Anna Lindstrand, Anna Wedell, Henrik Stranneheim

**Affiliations:** 1 Science for Life Laboratory, Department of Microbiology, Tumor and Cell Biology, Karolinska Institutet, Stockholm, Sweden; 2 Science for Life Laboratory, School of Engineering Sciences in Chemistry, Biotechnology, and Health, KTH Royal Institute of Technology, Stockholm, Sweden; 3 Department of Clinical Genetics, Karolinska University Hospital, Stockholm, Sweden; 4 Department of Molecular Medicine and Surgery, Karolinska Institutet, Stockholm, Sweden; 5 Center for Molecular Medicine, Karolinska Institutet, Stockholm, Sweden; 6 Centre for Inherited Metabolic Diseases, Karolinska University Hospital, Stockholm, Sweden

**Keywords:** Genomics, Rare disease, Mendelian, Single nucleotide variant, Structural variant, Population frequency

## Abstract

**Background:**

Exome and genome sequencing is becoming the method of choice for rare disease diagnostics. One of the key challenges remaining is distinguishing the disease causing variants from the benign background variation. After analysis and annotation of the sequencing data there are typically thousands of candidate variants requiring further investigation. One of the most effective and least biased ways to reduce this number is to assess the rarity of a variant in any population. Currently, there are a number of reliable sources of information for major population frequencies when considering single nucleotide variants (SNVs) and small insertion and deletions (INDELs), with gnomAD as the most prominent public resource available. However, local variation or frequencies in sub-populations may be underrepresented in these public resources. In contrast, for structural variation (SV), the background frequency in the general population is more or less unknown mostly due to challenges in calling SVs in a consistent way. Keeping track of local variation is one way to overcome these problems and significantly reduce the number of potential disease causing variants retained for manual inspection, both for SNVs and SVs.

**Results:**

Here, we present loqusdb, a tool to solve the challenge of keeping track of any type of variant observations from genome sequencing data. Loqusdb was designed to handle a large flow of samples and unlike other solutions, samples can be added continuously to the database without rebuilding it, facilitating improvements and additions. We assessed the added value of a local observations database using 98 samples annotated with information from a background of 888 unrelated individuals.

**Conclusions:**

We show both how powerful SV analysis can be when filtering for population frequencies and how the number of apparently rare SNVs/INDELs can be reduced by adding local population information even after annotating the data with other large frequency databases, such as gnomAD. In conclusion, we show that a local frequency database is an attractive, and a necessary addition to the publicly available databases that facilitate the analysis of exome and genome data in a clinical setting.

## Background

Knowledge about population frequencies is of utmost importance when deducing pathogenicity of genetic variants in individuals with rare disorders. In recent years, advancements in high-throughput sequencing have allowed for comprehensive sequencing of many individuals from various populations. The accumulated data is publicly available in a number of databases that describe the background variation in the general population as well as in some subpopulations. ExAC [1] and gnomAD [2] are the most comprehensive catalogs of human variation available today and are often used to filter out benign background variation from whole exome (WES) and genome (WGS) sequencing data. However, most such efforts are focused on single nucleotide variants (SNVs) and small insertions and deletions (INDELs) and knowledge is still limited when it comes to larger structural variation (SV) background frequencies. Existing resources include the Database of Genomic Variants (DGV) [3], Decipher [4] and the more recently published gnomAD SV track [5]. It is therefore of interest for sequencing centers and clinical labs to keep track of variants that are observed, especially when working in a setting where hundreds or thousands of genomes and exomes are sequenced every year. Many laboratories have their own solution to study the local variation that stems both from local population frequencies and sequencing artifacts [6]. There are some public tools available, such as the gemini framework [7] and Leiden Open Variation Database (LOVD) [8], to build a local variation database. Some of these tools, for instance LOVD, are missing support for the more complex SVs and others, e.g. gemini, are cumbersome to update continuously since the whole database needs to be rebuilt every time a new sample is added. Here we present **loqusdb**, a solution to the challenge of handling local variant observations for both SNVs/INDELs and SVs. To study the value of a local observations database, variants from a publicly available dataset of 1000 Swedish individuals (SweGen cohort) [9] were used to simulate the scenario in a diagnostic lab. By filtering the generated data versus databases of different size and sets of genes with known disease association (gene panels) we show that the number of potential disease causing variants is significantly reduced compared to filtering using variant frequencies in gnomAD alone. Furthermore, we show that the size of the local database is directly proportional to the number of variants remaining after filtering. Strikingly, filtering using smaller databases (50 samples) provides direct clinical value as this remove 87% of common and local variants.

## Implementation

Loqusdb is implemented in python with a mongoDB backend (https://www.mongodb.com/) and operates on files in the standardised variant calling file (VCF) [10] and pedigree (PED) (http://zzz.bwh.harvard.edu/plink/data.shtml#ped) format. Variant calls from short read data is for traditional and computational reasons divided into three broad categories: small variants SNV (single nucleotide variation) or INDEL (insertion and/or deletion of a few base pairs, typically contained within the read length) and SV deletion (DEL), duplication (DUP), insertion (INS), inversion (INV), break-end (BND), with the latter allowing for translocation, transposition. While these categories are somewhat arbitrary, the database tool makes no further attempts to unify or subdivide these, but relies on variant callers to make the distinction. Loqusdb will assume that all variant records are decomposed into single alternative alleles and normalized according to best practices, preferably by using VT [11]. We denote a family or a single individual upload a case. Each variant line in a VCF will only be counted as one observation, regardless of the number of individuals in the case that carry the variants. Loqusdb is a tool developed for a clinical setting, it is expected that the variant information originates from probands and carriers in the form of patient family members. To avoid enrichment of pathogenic variants due to a higher proportion of affected individuals than in the general population each observed variant is counted once for every family regardless of the number of carriers. If the variant is called homozygote alternative in any of the individuals this information of also saved to the database. Cases are loaded via a command line interface (CLI) with the command:


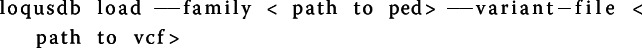


As a default, variants with a genotype quality (GQ) higher than 20 are loaded to ensure that only high quality variant calls are stored. This setting can be modified or set to zero if all variants should be included. Loqusdb checks if the case already exists in the database, either by case id or a sample ID profiling feature (described in Supplementary Materials section Sample ID profiling, Additional file 1). The variant insertion process will only start if the case did not previously exist in the database. Note that unlike other frequency databases loqusdb can be updated continuously and does not need to be rebuilt each time a new case or batch of cases are added.

### SNVs

For SNVs and small INDELs a unique id is created for each variant by concatenating chromosome, position, reference allele and alternative allele into a string. This string is used as a “primary key”, or “document id” in mongoDB, to search for a variant. When a variant is added to the database we first check if the variant exists, in this case the observations counter is incremented by one. If the variant is observed in a homozygous state in any of the individuals of the family the homozygous counter is also incremented with one. Finally, the case id is added to the list of cases on the variant, making it easy to track if the variant has been seen in any relevant cases. If the variant has never been seen before it is added with counters and variables set according to the description above.

### SVs

**Fig. 1:**
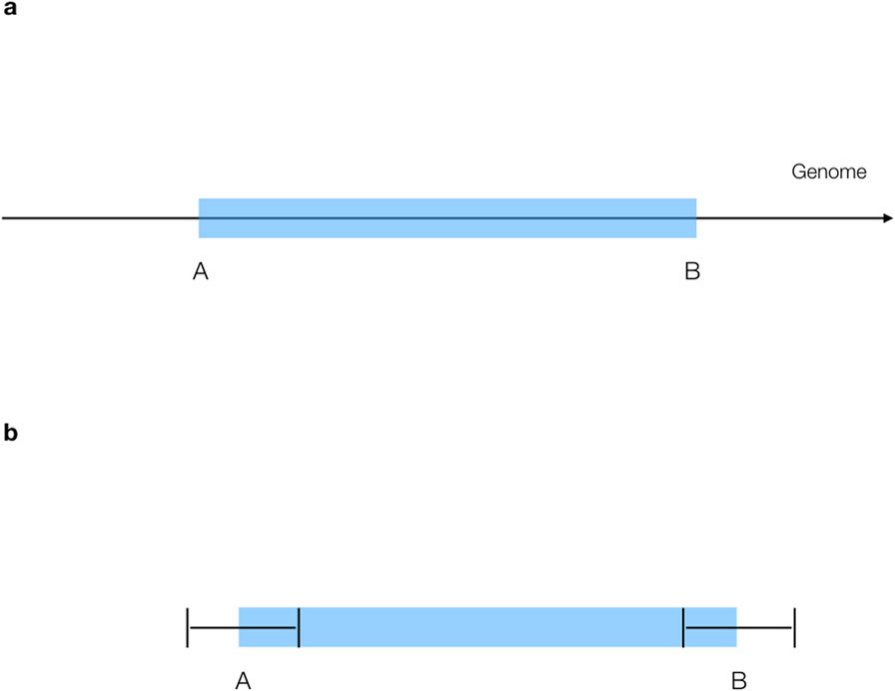
Illustration of SV representation in loqusdb. Illustration of a structural variant **a** where A and B are the start and end coordinates. **b** visualizes how the same SV is represented in loqusdb with dynamic intervals around each endpoint. Size of intervals will vary with size of SV

Loqusdb defines each SV by two properties: the SV-type and position. The SV-type is extracted from the ALT column of the input VCF file, and include deletion, duplication, and inversion - as well as any other callable SV type following the VCF specification. The position of the SV is defined as a set of two coordinates, position A and position B (Fig. 1a); each coordinate consists of a the chromosome name and the genomic position. Together these represent the start and end positions of the SV. For *intrachromosomal* variants, the chromosome of position A and B are the same, and the genomic positions are set to the start and end positions of the SVs. For *interchromosomal* variants, position A is set to the chromosome and position reported in the CHROM and POS columns of the VCF file, while position B is set to the chromosome and position reported in the ALT column, all according to standards in the VCF format. Some SVs, such as inversions and translocations, are often represented as two events in the VCF file. As the purpose of loqusdb is to identify recurring events, these variants are not treated different than any other type of variant. This is based on the assumption that variant callers will represent biological events in the same way even if they are called in different individuals. SV calling is complicated, and the reported breakpoint positions of an SV are sometimes not precise, due both to variability in variant calling and biological processes. Hence the position of the same SV may differ between samples and callers. Loqusdb addresses this issue by generating clusters of similar SVs. The number of individuals present in each cluster are used to estimate the allele count of the SVs. These clusters are generated or updated on the fly as SVs are added to the database. Similar to an SV, a cluster is defined as an SV-type and a set of two coordinates, additionally an interval is added around the positions of a cluster to account for imprecision(Fig. 1b). Per default, the length of the interval is set to 2 kb for variants larger than 10 kb, and 10% of the variant length for smaller variants. An interchromosomal variant is always treated as a variant larger than 10 kb (since its length is infinite). The length of an interval needs to be long enough to cover different representations of SVs due to the noise of the sequencing data; yet short enough to not place variants that differ too much in the same cluster. Users may fine-tune these settings to fit various data sources, variant callers, and purposes of the database. If an SV is added to the database, loqusdb will search for a matching cluster. An SV matches a cluster if their SV-type is the same, and the positions of the SV is within the interval of the cluster. If the SV does not match any cluster within the database, a new cluster will be formed. This cluster will have the same type and position as the newly added SV, and an interval as explained previously. If the SV matches an existing cluster, the SV will be added to that cluster. Once the variant is added to the cluster, the positions of the cluster will be updated to the average position of the SVs belonging to the cluster. This means that the size of the endpoint intervals remains the same. If an SV matches multiple clusters, it will be added to the closest cluster only.

## Annotation and export

Loqusdb offers two ways of annotating variants: (1) by exporting all variants to a VCF including the observations for use with annotation tools such as vcfanno [12] or (2) through the CLI with simple commands (described in Supplementary material section *Annotation of local observations*, Additional file 1). When annotating SVs the same algorithm described above “SVs” section when adding SVs is used to determine if a structural variant matches any of the existing ones in the database.

## Results and discussion

To illustrate the utility of loqusdb we used variants from the SweGen cohort [9], a public dataset consisting of 1000 Swedish individuals chosen to maximise the representation of genetic diversity in the Swedish population. PCR-free WGS libraries (Illumina TruSeq DNA PCR-free) were prepared from blood samples and sequenced on the Illumina Hiseq X platform, producing on average 36X coverage per individual; the libraries were sequenced to a read length of 2x151 base pairs. The sequencing dataset was preprocessed (Supplementary materials sections *Variant calling* and *Data processing, analysis and filtering*, Additional file 1) and divided into two parts where 98 samples were randomly sampled and separated from the rest. The remaining 888 individuals were used to generate a local observations database of genomic variation using loqusdb (Supplementary Methods section *Construction of Local Databases*, Additional file 1). The 98 individuals not included in the background database were used for various filtering scenarios. Fourteen individuals were excluded due to problems during preprocessing, such as segmentation faults while running the copy-number analysis using CNVnator version 0.3.3 [13].

### Local observation database versus gnomAD

**Fig. 2:**
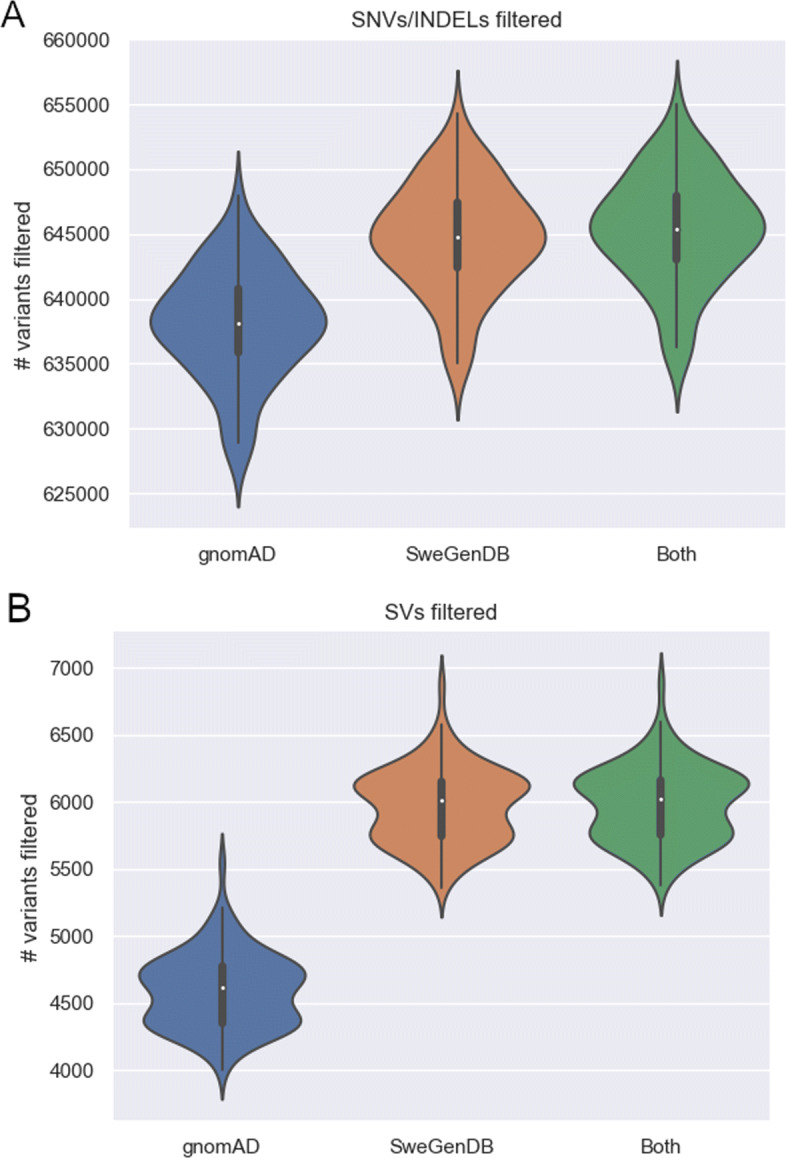
Comparison of a local frequency database versus gnomAD. Violin plots presenting the number of filtered variants using a local frequency database (SweGenDB) versus a public database (gnomAD). The local frequency database consists of 888 individuals. The frequency filter was applied on SNV calls **a**, and SV calls **b**

All variants identified in 98 unrelated individuals were annotated using the gnomAD frequency database as well as SweGenDB. Variants with an AF higher than 1% were removed, but the variant filtering was performed with three separate combinations of frequency databases: (1) only gnomAD; (2) only SweGenDB; (3) any of the two databases (Fig. 2). The SweGenDB filtered out a larger number of variants compared to gnomAD. Additionally, no significant advantage was found when considering both databases simultaneously compared to SweGenDB alone (Figs. 2a, 2b). These results were true for both SNVs (Fig. 2a) and SVs (Fig. 2b). However, the local SV database filters out 30% more variants than gnomAD (mean number of variants removed was 4591 for gnomAD and 5971 for SweGenDB). In contrast, the gnomAD and local SNV databases are more similar, with the local database filtering on average only 1% more SNVs than gnomAD (mean 638091 for gnomAD and 644737 for SweGenDB). SV callers are known to be sensitive to technical artifacts, including read depth variation and GC% bias. The reason why gnomAD performs more similar to SweGenDB on SNV/INDEL compared to SV may therefore be due to local technical artifacts, use of different SV callers and differences between the dataset used by gnomAD and SweGenDB. Notably, the number of filtered SVs follows a bimodal distribution, while the amount of filtered SNVs follows a unimodal distribution (Figs. 2a, 2b). The bimodal SV distribution indicates that there are two groups of samples: one group that is more similar to the individuals in the databases (SweGenDB, and gnomAD), and one group that is relatively dissimilar. Analyzing the chromosome Y haplogroups of all male individuals, we found that the number of filtered SV calls vary among Y haplogroups (Supplementary material Figure 1, Additional file 1 and Table 1, Additional file 2). In particular, the distribution of filtered SV follow unimodal distributions in the majority of common (>10 individuals) chromosome Y haplogroups groups (14/16) (Supplementary material Figure 1, Additional file 1), and the haplogroups: i1a2a and R1b1a1b1a1a, are the only common haplogroups that follow clear bimodal distributions. Next, the chromosome Y haplogroups were grouped into 10 major groups: E1, G2, I1, I2, J2, N1a1, Q1, Q2, R1a, and R1B (Table 2, Additional file 3). We compared the number of filtered SV per individual in these groups and find significant differences; in particular, we found that individuals carrying haplogroup I1, a haplogroup originating in Europe [14], are filtered more efficiently (p=0.045) compared to individuals carrying N1a1 - which is a haplogroup common in Finland and the Baltic region [15].

#### Gene panels

**Fig. 3:**
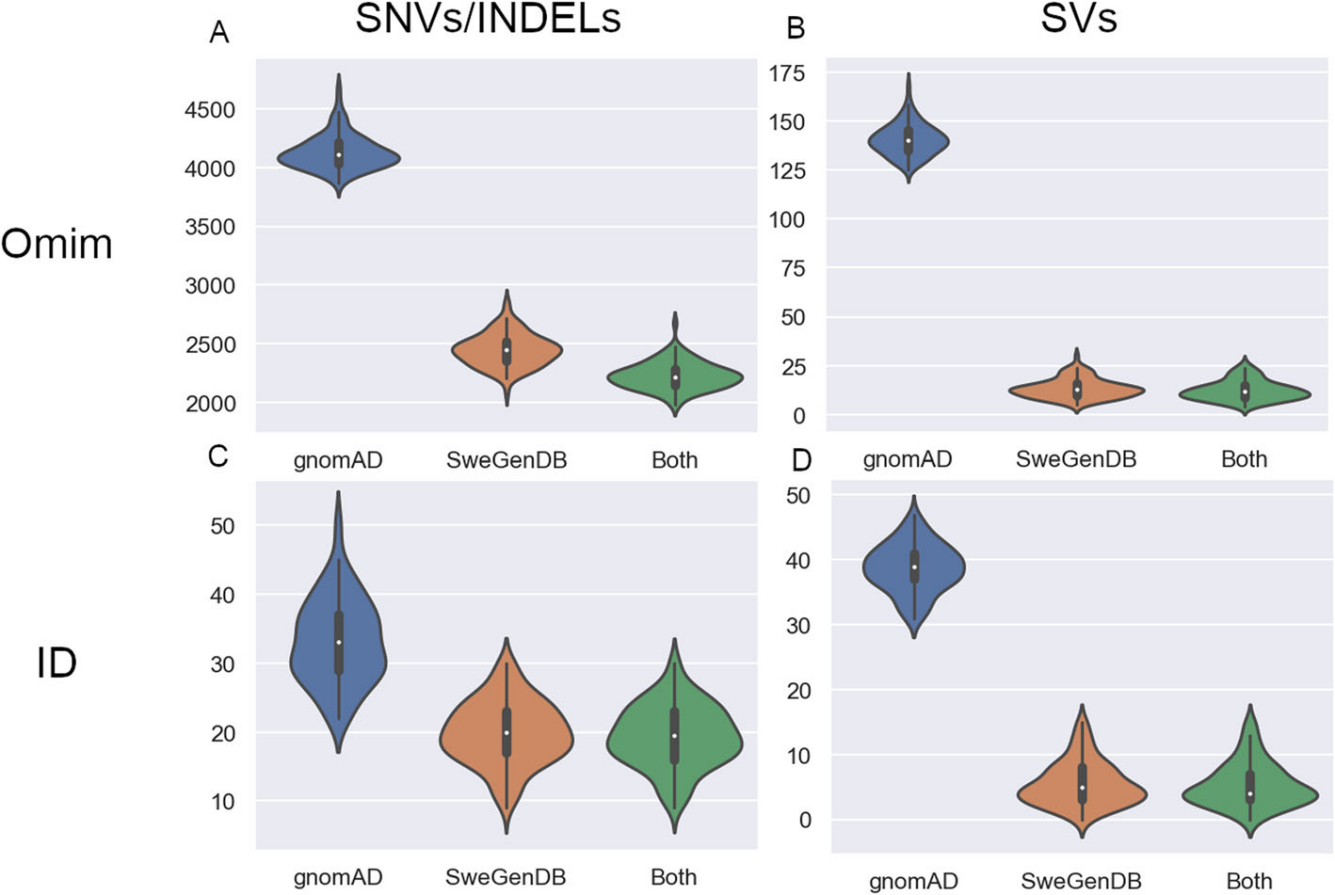
Comparison of variants remaining between gnomAD vs. SweGenDB based filtering after application of in-silico gene panels. The number of rare SNVs **a**, and rare SV **b** in the OMIM gene panel remaining after filtering. The number of rare and “HIGH” consequence SNVs **c**, and SV **d** in the Panel App Intellectual disability panel remaining after filtering

To simulate the situation used in a clinical setting, we chose to filter all variants with frequency below 1% for two in silico gene panels: the PanelApp intellectual disability (ID) gene panel, and the OMIM panel (≈3800 genes) as described in Supplementary Materials section *Data processing, analysis and filtering*, Additional file 1. To make this example more clinically relevant we chose to include variants with “HIGH” impact according to VEP. An impact of “HIGH” is assigned for any of a set of consequence terms that are predicted to severely impact the transcript it affects, such as frame-shift mutation or transcript ablation. For both panels, twice as many variants remained using gnomAD compared to SweGenDB (*p*<<0.01; T-test) (Fig. 3). Filtering for the combination of gnomAD and SweGenDB was more effective than using only one of the databases (*p*<<0.01 for both vs gnomAD and *p*<<0.01 for both vs SweGenDB; T-test). Notably, there was a small advantage in filtering SNVs exceeding the AF threshold in any of the databases (Figs. 3a, 3c). However, such an advantage was not found among the SVs (Figs. 3b, 3d). Interestingly, the rare SV count distributions in panels were unimodal, which was not the case for the SV count distribution across the entire genome (compare Fig. 2b).

#### Sizes of database

**Fig. 4:**
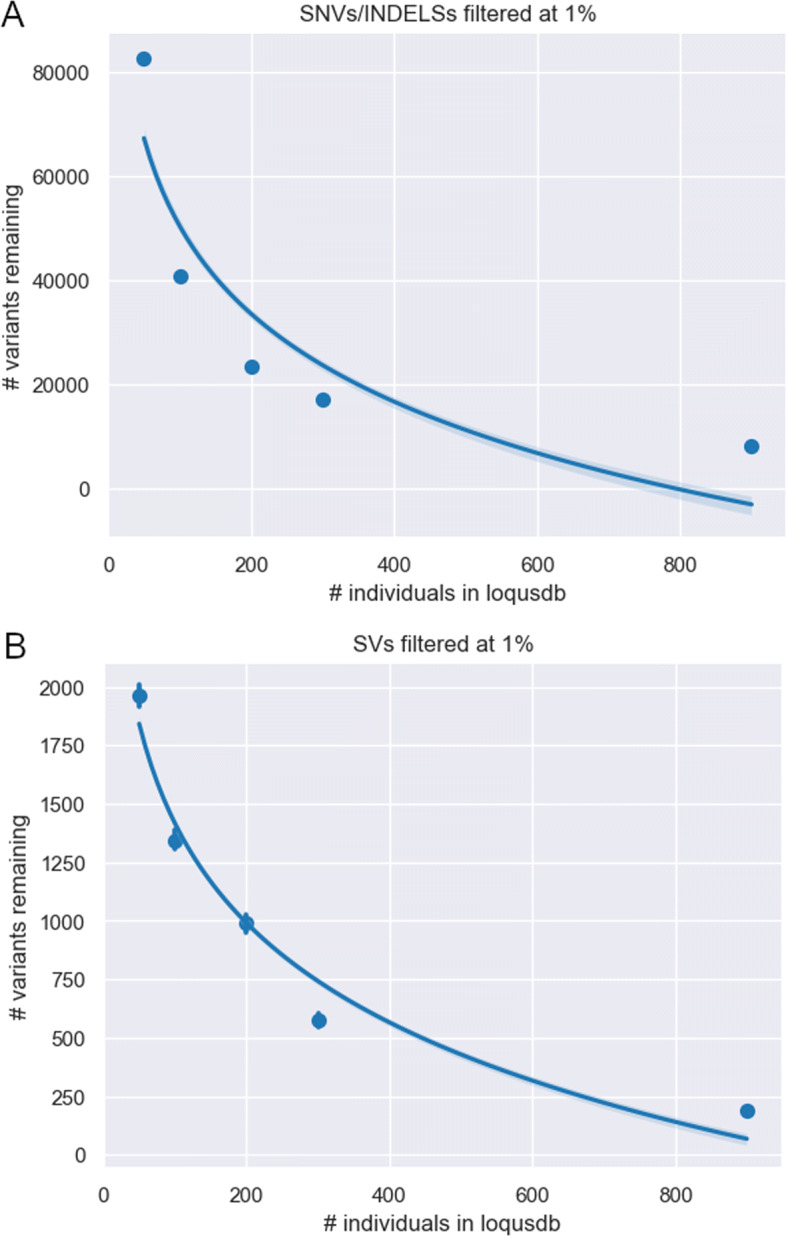
The size and effect of a local frequency database. The average number of rare SNVs **a** and SV **b** per individual using databases of different sizes

Next we assessed how the size of a database affects the efficiency of variant removal. To study this we created databases of different sizes, ranging from 48 to 888 individuals. We then used the established database to annotate observed frequencies for the variants in the 98 test individuals, that were not included in any of the databases. The results showed a logarithmic relationship between the number of rare calls remaining after filtering and the size of the database. A database with 48 individuals removes the majority of SNVs (87%) and SVs (70%), while the largest database possible in this setting (888 individuals) filters out almost 99% of the SNVs and 97% of the SV (Figs. 4a, 4b). Furthermore, a database of 888 individuals removes nearly twice the number of variants as the database of 296 individuals (Fig. 4). These results indicate that relatively small number of individuals in a local observation count database (≈50 individuals) is useful for separating rare from common variants, but also that there is added value in constructing databases with many individuals (≈900 individuals).

## Conclusions

We present a novel tool, loqusdb, to solve the challenge of maintaining a local, real-time updated, observation count database for both SNVs, INDELS and SVs. Using a publicly available dataset, we show that a local observations database identifies a significantly larger number of rare variants (AF <1%) compared to the largest public dataset available (gnomAD), thus reducing the number variants remaining for manual triage in a clinical setting. Furthermore, we show that the value of the database scales with the size; but also that databases with a fairly low number of individuals are useful in filtering normal local variation and systematic artefacts.

## Supplementary information

**Additional file 1** Supplementary material. This file includes the supplementary material. File is in pdf format.

**Additional file 2** Table S1. SV variants filtered and haplogroup for each individual. File is in csv format.

**Additional file 3** Table S2. SV variants filtered for major haplogroups. File is in csv format.

## Abbreviations

SNV: Single nucleotide variant
INDEL: Small insertion and deletion variants, with precise start and stop positions
SV: Structural variant, larger genomic variations that often have imprecise start and stop positions
VCF: Variant call format, standardised file format used for genomic variation
GQ: Genotype quality, a measurement of the certainty of a genotype call
WES: Whole exome sequencing
WGS: Whole genome sequencing
gnomAD: Genome aggregation database, public resource including >15K WGS and 120K WES; https//gnomad.broadinstitute.org
DGV: Database of Genomic Variants, used for structural variants
SWEGEN: Public resource with 1000 unrelated Swedes from the national twin registry
SweGenDB: An instance of loqusdb with local observations from the Swedish population
VEP: Variant effect predictor - a tool to annotate variants in the VCF format
OMIM: Online inheritance in man (https://omim.org)
CLI: Command line interface
