# Supplementary material for: Loqusdb: added value of an observations database of local genomic variation
Source: BMC Bioinformatics. 2020 Jul 1;21:273. doi: 10.1186/s12859-020-03609-z (PMC7329469; doi:10.1186/s12859-020-03609-z)
Supplement: Supplementary file 1 — Additional file 1 Supplementary material. This file includes the supplementary material. File is in pdf format. [file 12859_2020_3609_MOESM1_ESM.pdf]

Supplementary Materials

Variant calling

For SNVs/INDELs the sequencing data were preprocessed and analyzed according to the standard GATK best practice procedure [1]. More details about the data processing is described in [2]. The SNV files were then decomposed and normalized with VT version 0.5772 (`https://genome.sph.umich.edu/wiki/Vt`). Structural variants were generated using findSV (`https://github.com/J35P312/FindSV`), a pipeline that combines output from the SV callers TIDDIT 2.2.6 [3] and CNVnator version 0.3.3. FindSV was executed using the binary alignment (BAM) files from the preprocessing stage described in the paragraph above. We chose to only include variants from confidently callable parts of the genome as described in [4] to avoid problematic regions, often due to low complexity, with uncertain variant calls.

A. Data processing, analysis and filtering

First, a local database, SweGenDB, was constructed from variants detected in 888 individuals from the SweGen cohort by loading their data into a local loqusdb version 2.4 database (see Supplementary section: Construction of Local Databases D). We proceeded with the annotation of variants detected in 98 other SweGen samples, using allele frequencies from gnomAD version 2.1.1. SNVs/INDELs were annotated with with VEP v92 [5], SVs were annotated with SVDB (`https://github.com/J35P312/SVDB`) (see Supplementary Materials E and F for more details). The variants were then also annotated with the observed number of occurrences (observations) per alternative genotype in SweGenDB, both for SNVs/INDELs and SVs (see Supplementary Materials G for more details). Next, we performed different filtering scenarios to compare the number of variants that could be dismissed based on frequency compared to gnomAD, SweGenDB or both. For all scenarios, we removed variants with allele frequencies (AF) higher than 1% according to gnomAD VCF key POPMAX_AF. Sets of genes with known or suspected association with disease were collected into gene panels based on disease phenotypes. This is a common approach to reduce the number of variants under investigation to a relevant clinical subset of candidate variants. The variable measured was the number of variants left to interpret scaled by different sizes of gene panels. We used the PanelApp intellectual disability gene panel version 2.833 (979 consensus genes) (ID) from the PanelApp (`https://panelapp.genomicsengland.co.uk`), and a Mendeliome panel consisting of all disease associated genes in the Online Mendelian Inheritance in Men (OMIM) (`https://omim.org/`; 3756 genes). In the OMIM panel all genes where a disease relationship is "established" or "provisional" was included. We also investigated how the number of individuals in a local observation database affects the number of variants that can be dismissed. All experiments were performed for both SVs and SNVs/INDELs.

B. Chromosome Y haplogroups

The chromosome Y haplogroups were predicted using Yleaf 2.0 [6]. Yleaf was run using the following command:

```
Yleaf.py -bam $1 -r 10 -q 30 -t 6 -out $2 -ref hg19 -b 95
```

Were $1 is the WGS bam file, and $2 is the output file. The command was run for all individuals in the SweGen cohort. Individuals having a non-zero Q score was considered male, all other individuals were assumed to be female; statistical assessments were performed using the Mann Whitney U test. Results are visualised in Figure 1 and Tables I and II.

C. Sample ID profiling

Loqusdb has a genotype-based sample ID profiling feature to detect if a case has already been inserted into the database. This is to avoid sample duplication in the database, which will have negative effects on the observation counts. The user provides a set of positions, each case will then get a combined mutation profile depending on what variation they have. These variants should be normal variants in the population and chosen so that each combination is unique. Loqusdb comes with a standard set of 50 SNVs that can be used as a default set of positions for sample profiling. When a new case is loaded - loqusdb will create a string based on the variant calls of these positions and look at the hamming distance with all existing cases in the database. For more information see loqusdb documentation.

D. Construction of Local Databases

Local databases were constructed using loqusdb version 2.4 (https://github.com/moonso/loqusdb). The databases were constructed using the following command:

```
loqusdb --port $PORT load --variant-file $SNV_VCF --sv-variants $SV_VCF --gq-treshold 0 -
```

Were SNV_VCF is a SNV VCF file, SV_VCF is a SV VCF, PORT is the port used by the loqusdb instance, SAMPLENAME is the name of the sample. The command is run once for each individual to be loaded into the database. This command was used to construct six databases of different size (10, 48, 100, 196, 296, 888 individuals respectively); these databases were subsequently used to annotate the VCF files of 98 individuals not included in any of the databases.

E. Annotation of SNV VCF files

The resulting VCF files were annotated using the following VEP command:

```
vep -i $TMPDIR/tmp.vcf -o $TMPDIR/vep.vcf --assembly GRCh37 --cache --offline --vcf --for
```

This command performs annotation using VEP, and adds the gnomAD database as a custom annotation using the --custom flag. The gnomAD VCF was downloaded from the following site: https://storage.googleapis.com/gnomad-public/release/2.1.1/vcf/genomes/gnomad.genomes.r2.1.1.sites.vcf.bgz. The annotated VCF files were subsequently filtered to only include variants from Genome in a bottle high confident regions (ftp://ftp-trace.ncbi.nlm.nih.gov/giab/ftp/release/NA12878_HG001/latest/GRCh37/supplementaryFiles/HG001_GRCh37_GIAB_highconf_CG-IllFB-IllGATKHC-Ion-10X-SOLID_CHROM1-X_v.3.3.2_highconf.bed) using bedtools v2.27.1 with the following command:

```
bedtools intersect -header -a $TMPDIR/vep.vcf -b HG001_GRCh37_GIAB_highconf_CG-IllFB-IllGA
-u -wa | grep -E "#|LOW|HIGH|MODERATE" > $1.filt.vcf
```

Additionally, this command filters for variants having a low, moderate, or high consequence (https://www.ensembl.org/info/genome/variation/prediction/predicted_data.html). The resulting filtered VCF file was annotated using each one of the internal frequency databases.

F. Annotation of SV VCF files

SVDB 2.0.0 (https://github.com/J35P312/SVDB) was used to annotate the gnomAD frequencies, and was run using the following command:

```
svdb --query --query_vcf $SV_VCF --db gnomad_popmax.vcf --frequency_tag POPMAX_AF --hit_t
```

The gnomad_popmax.vcf file was produced by selecting all variants containing the POPMAX_AF entry from the gnomad_v2_sv.sites.vcf.gz:

```
zgrep -E "POPMAX_AF|#" gnomad_v2_sv.sites.vcf.gz > gnomad_popmax.vcf
```

This was done to remove variants lacking the POPMAX_AF sites, as SVDB requires all variants to carry the frequency information specified by the --frequency_tag parameter.

G. Annotation of local observations

Finally all VCFs, both SNVs and SVs, where annotated with the local observations from the Loqusdb instance with the command:

```
loqusdb -port $PORT annotate $SNV_VCF > $FOLDER/$SNV_FILE
```

for SNVs, and:

```
loqusdb -port $PORT annotate --sv $SV_VCF > $FOLDER/$SV_VCF
```

for SVs.

Where SNV_VCF is a SNV VCF file, SV_VCF is a SV VCF, PORT is the port used by the mongoDB instance, FOLDER is the folder that the annotated file will be printed to. These two commands are run separately for each individual and database size.

H. Variable tresholds

The treshold of 1% is used in the article since this is the most commonly used definition of a rare variant. The plots below show that the relation between the local database and gnomAD is persistent with different tresholds. We show results for 1% (Figure 2), 0.5% (Figure 3) and 0.25% (Figure 4)

---

[1] M. A. DePristo, E. Banks, R. Poplin, K. V. Garimella, J. R. Maguire, C. Hartl, A. A. Philippakis, G. del Angel, M. A. Rivas, M. Hanna, et al., Nature Genetics **43**, 491 (2011), ISSN 1546-1718.

[2] A. Ameur, J. Dahlberg, P. Olason, F. Vezzi, R. Karlsson, M. Martin, J. Viklund, A. K. Kähäri, P. Lundin, H. Che, et al., European Journal of Human Genetics **25**, 1253 (2017), ISSN 1018-4813, 1476-5438.

[3] J. Eisfeldt, F. Vezzi, P. Olason, D. Nilsson, and A. Lindstrand, F1000Research **6**, 664 (2017), ISSN 2046-1402.

[4] J. M. Zook, B. Chapman, J. Wang, D. Mittelman, O. Hofmann, W. Hide, and M. Salit, Nature Biotechnology **32**, 246 (2014), ISSN 1087-0156, 1546-1696.

[5] W. McLaren, L. Gil, S. E. Hunt, H. S. Riat, G. R. S. Ritchie, A. Thormann, P. Flicek, and F. Cunningham, Genome Biology **17**, 122 (2016), ISSN 1474-760X.

[6] A. Ralf, D. Montiel González, K. Zhong, and M. Kayser, Molecular Biology and Evolution **35**, 1291 (2018), ISSN 0737-4038.

Tables

TABLE I: Table S1. SV variants filtered and haplogroup for each individual.

TABLE II: Table S2. SV variants filtered for major haplogroups.

Figures

S1.png

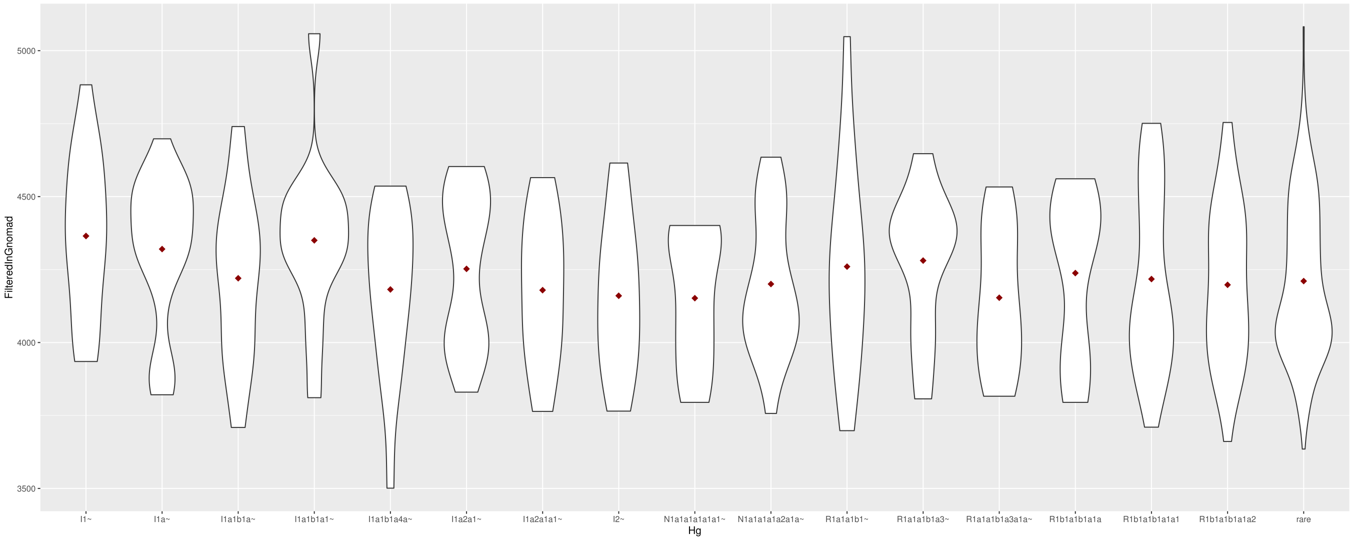

FIG. 1: SV variants filtered depending on haplogroup.

S2.png

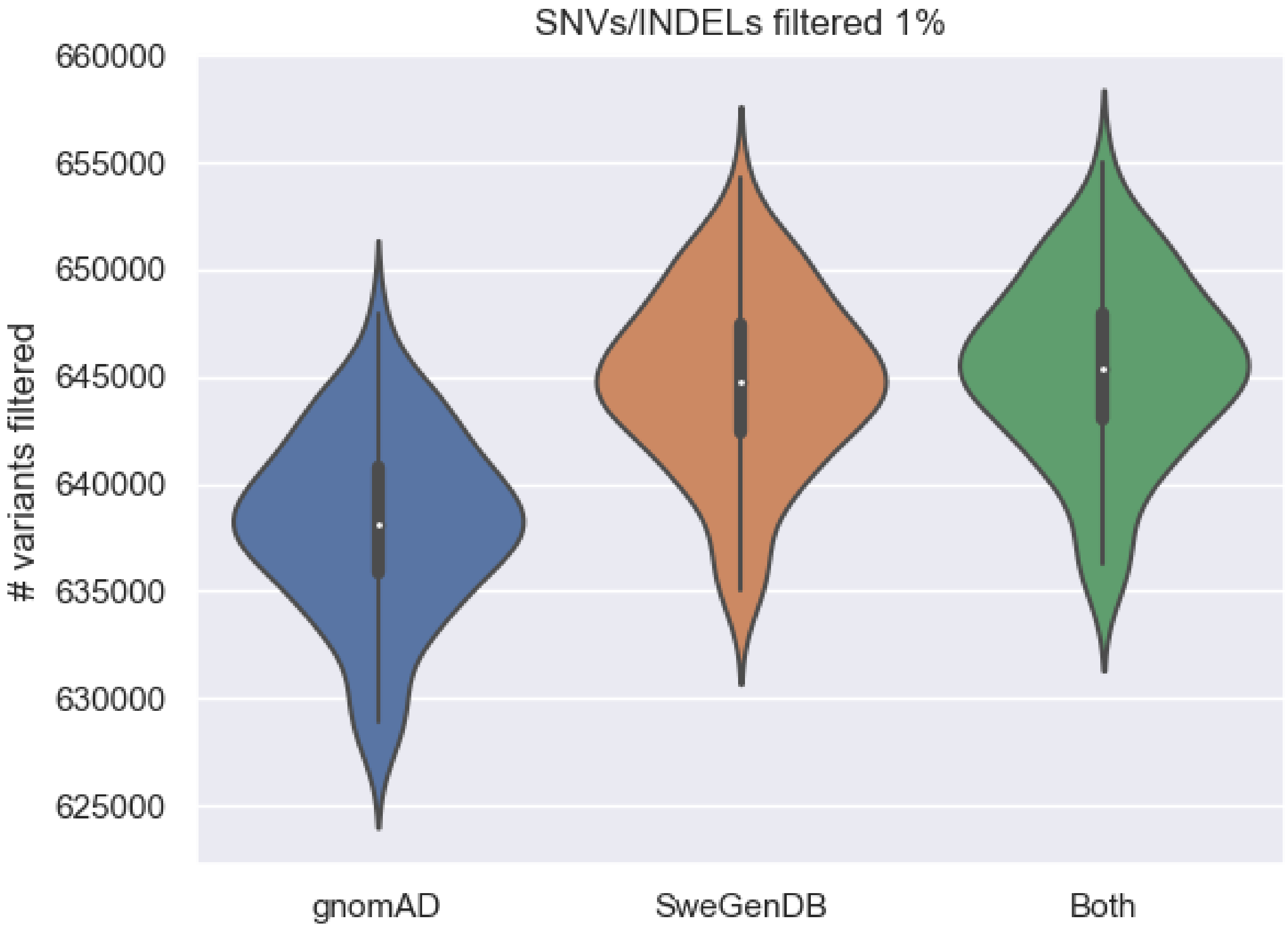

FIG. 2: Variants filtered at 1% treshold.

S3.png

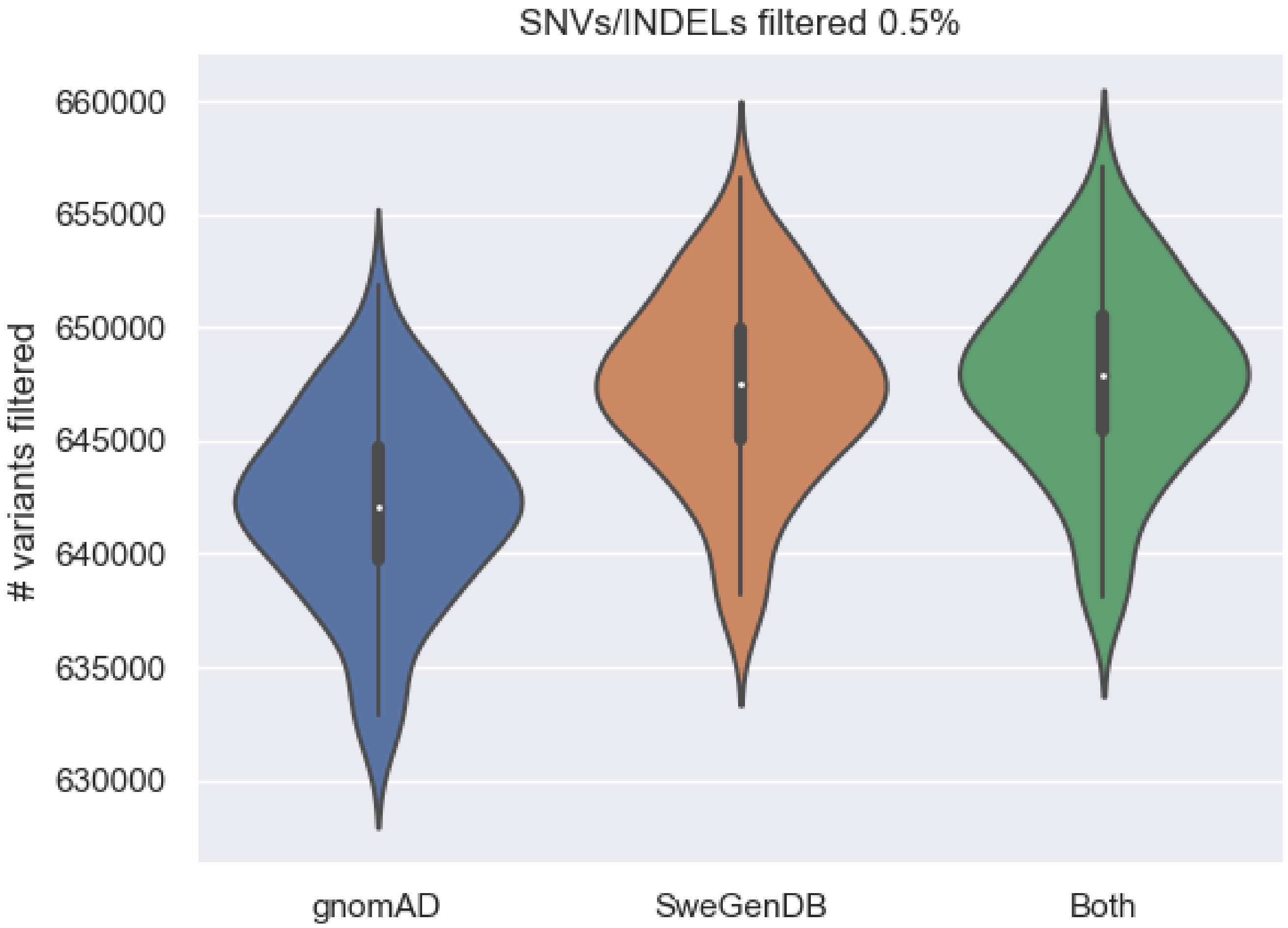

FIG. 3: Variants filtered at 0.5% treshold.

S4.png

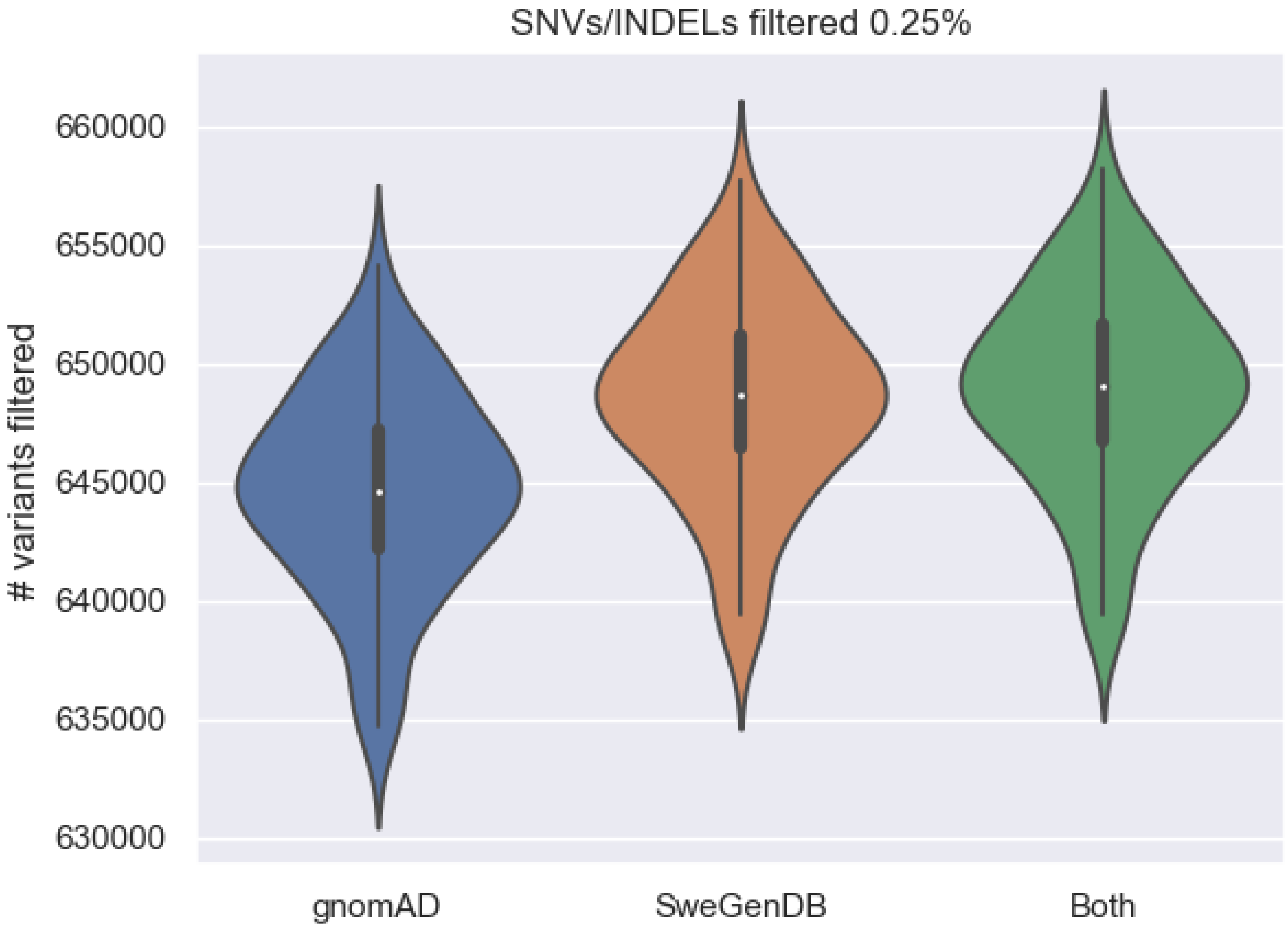

FIG. 4: Variants filtered at 0.25% treshold.
